# Profile of Geriatric Patients Attending the Emergency Department of a Tertiary Care Hospital in Karachi: A Cross-Sectional Study

**DOI:** 10.7759/cureus.21874

**Published:** 2022-02-03

**Authors:** Saima Mushtaq, Muhammad Tarish Abro, Muhammad Sualeh, Muhammad Roohan Uddin

**Affiliations:** 1 Internal Medicine, Jinnah Postgraduate Medical Center, Karachi, PAK; 2 Department of Medicine, Jinnah Sindh Medical University, Karachi, PAK

**Keywords:** charlson comorbidity index, co-morbidities, frailty, emergency department, geriatrics

## Abstract

Background and Objectives

The emergency department (ED) is the most important unit of a hospital and is often visited by an ever-increasing proportion of geriatric patients. However, in Pakistan, there is an inadequacy of geriatrics specialists. The objective of this study was to assess the profile of geriatric patients in the ED. We considered main diagnoses, frailty, and other factors that help to come up with certain findings that may assist with policymaking and initiatives for better geriatric care in Pakistan.

Methods

A cross-sectional study was conducted at a hospital in Karachi, Pakistan. The study population comprised 101 patients aged 65 years and older who attended the hospital’s ED from July to September 2021. Data were collected by taking the patients’ histories.

Results

The majority of patients were aged between 65 and 70 years. The most common diagnoses were: gastrointestinal disorders, cerebrovascular accidents, and neurologic disorders. More than half of the patients were found to be frail by the five-item FRAIL questionnaire. The mean Charlson Comorbidity Index score was 5.37 ± 1.88.

Conclusion

It was concluded that the need for geriatric care is worth mentioning since it imposes a significant burden on the ED. The geriatric patients had a higher risk of being on the critical list as most of them were frail and suffered from multiple severe comorbidities. Our results can assist in the development of geriatric emergency medicine and acute care systems in Pakistan.

## Introduction

The geriatric population is growing very rapidly, which is of increasing concern worldwide. Pakistan’s 2017 national census reported a population of 200 million, the sixth-highest in the world, of which about 13.7 million (7%) are aged 60 years and older [1]. This proportion of the population is expected to increase to 12% by 2050 [2]. Such a vast geriatric population requires the health care system to provide proper resources and services.

The emergency department (ED) is the most important unit of a hospital because any dysfunction in the ED results in the whole systemic dysfunction [3]. There is an increasing proportion of ED visits by elderly people [4] due to increasing and multiple comorbidities, increasing socioeconomic and financial pressures, and limited health care resources. In Pakistan, there is an inadequacy of specialists in the field of geriatrics. Consequently, geriatric care is often handled by general practitioners or specialists in other fields [5]. Furthermore, geriatric patients’ illnesses are often treated separately, without treating the whole patient [5]. It is important to address the needs of geriatric patients as they can undergo severe illnesses or injuries and arrive at the ED with multiple and complex problems [6]. Since older patients are more likely to use ED services than younger patients [7], we aimed to discover more about the profile of geriatric patients at the ED, including determining the most common diagnoses, frailty, and other factors. This may help with policymaking and taking appropriate actions for better geriatric care in Pakistan.

## Materials and methods

Design and setting

A descriptive cross-sectional study was conducted from July to September 2021 at the ED of Jinnah Postgraduate Medical Center, a tertiary care public hospital in Karachi. Most of its patients are of low socioeconomic status. The study was approved by the hospital’s Institutional Review Board.

Participants

Only participants who met certain criteria were included in the study. We included patients aged ≥65 years who presented to the ED during the above-mentioned period. We excluded patients who did not provide consent, those who presented without an attendant, and those who were dead on arrival.

Sample size

The study included 101 patients. The required sample size was calculated using the 7% proportion of ≥65-year-olds in the national population [1] with the following standard formula for calculating sample size based on prevalence. The margin of error was 5%, with a 95% confidence interval. No imputation method was used.

 $n=\frac{z^{2}\left \{ p\left ( 1-p \right ) \right \}}{d^{2}}$

Data collection and variables

Data were collected prospectively using a prestructured questionnaire. A pilot study was conducted to check the feasibility and validity of the questionnaire. All ethical considerations were observed. The questionnaire collected each patient’s sociodemographic data, main diagnosis, frequency of ED visits, and medical history. Frailty was determined using the five-item FRAIL questionnaire [8] developed by Maxwell CA et al., which addresses fatigue, resistance, ambulation, illness, and weight loss. These questions were answered based on the patient’s condition two weeks before admission. The FRAIL score ranges from 0 to 5, where 0 is robust, 1-2 is pre-frail, and 3-5 is frail. A score of one was assigned for every positive answer. The Charlson Comorbidity Index (CCI), developed by Mary Charlson et al., is used to predict the likelihood of death within one year of hospitalization for patients with specified comorbid conditions. Nineteen conditions are included in the index and are given points 1-6 based on the one-year mortality hazard ratio. By using the CCI calculator, the ten-year survival rate was also evaluated [9].

Statistical analysis

Data were analyzed using IBM Corp. Released 2017. IBM SPSS Statistics for Windows, Version 25.0. Armonk, NY: IBM Corp. Categorical variables were compared using the Chi-squared test. A p-value of <0.05 was considered statistically significant.

## Results

Data were collected from 101 patients (57 male, 44 female). Table 1 shows their sociodemographic data. The patients were divided into five subgroups by age. Most participants were in the 65-70 years age group.

**Table 1:** Patients’ sociodemographic data

	Male(n)	Female(n)	Total(n)	p-value
Age group				0.903
65-70	29	24	53	
70-75	11	6	17	
75-80	7	7	14	
80-85	4	2	6	
85+	6	5	11	
Education				0.004
Yes	26	8	34	
No	31	36	67	
Living status				0.630
Living alone	1	0	1	
Living with family	54	43	97	
Living with relatives	2	1	3	
Marital status				0.002
Single ​	1	2	3	
Married	37	13	50	
Widowed/divorced	19	29	48	
Ethnicity				0.682
Punjabi	4	3	7	
Sindhi	6	6	12	
Balochi	0	1	1	
Pashtun	7	8	15	
Urdu-Speaking	29	21	50	
Other	11	5	16	

**Figure 1:**
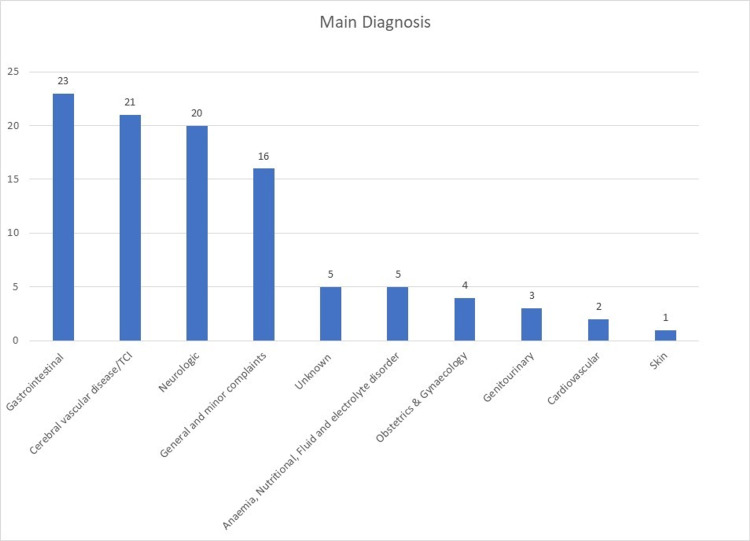
Main diagnosis

The patients’ most common diagnoses are shown in Table 1. The most common diagnoses were gastrointestinal disorders (n = 23), followed by cerebrovascular accident (n = 21), and neurologic disorders (n = 20). The gastrointestinal disorder was the most common diagnosis in both genders (p = 0.948) (Figure 1).

Fifty-six patients were frail (FRAIL score, 3-5), of which 28 were male, and 28 were female. Thirty-two patients were pre-frail (FRAIL score, 1-2), of which 21 were male, and 11 were female. Thirteen patients were robust (FRAIL score, 0), of which eight were male, and five were female (p-value = 0.336). The FRAIL questions that were answered positively were: loss of weight (n = 73), fatigue (n = 69), resistance (n = 60), ambulation (n = 51), and illness (n = 7). The mean CCI was 5.37 (standard deviation: 1.88). Common comorbidities included hypertension, history of falls, paralysis, and mental health problems. The most dominant comorbidity was hypertension (n = 58), followed by cerebrovascular accident (n = 37) and diabetes mellitus (n = 31) (Table 2).

**Table 2:** Comorbidities

	Male (n)	Female (n)	Total (n)	p-value
Charlson Comorbidity Index				
Cerebrovascular accident	18	19	37	0.150
Diabetes mellitus (uncomplicated)	13	15	28	0.134
Hemiplegia	13	14	27	0.161
Myocardial infarction	10	8	18	0.194
Dementia	7	8	15	0.179
COPD	5	7	12	0.135
Congestive heart failure	6	3	9	0.138
Chronic kidney disease (moderate to severe)	5	3	8	0.171
Peptic ulcer disease	3	4	7	0.167
Liver disease (severe)	2	4	6	0.118
Solid tumor (localized)	1	3	4	0.100
Diabetes mellitus (end organ damage)	2	1	3	0.177
Liver disease (mild)	3	0	3	0.05
COVID- 19	1	2	3	0.154
Solid tumor (metastatic)	2	0	2	0.080
Liver disease (moderate)	1	0	1	0.127
Peripheral vascular disease	0	0	0	0.072
Connective tissue disease	0	0	0	0.072
Leukemia	0	0	0	0.072
Lymphoma	0	0	0	0.072
AIDs	0	0	0	0.072
Other comorbidities				
Hypertension	33	25	58	0.914
Paralysis	16	16	32	0.374
Mental health problems	2	5	7	0.115
Others	5	6	11	0.437
History of falls	10	12	22	0.240
Estimated 10-year survival rate				0.642
0%	14	12	16	
2%	7	8	15	
21%	12	12	24	
53%	11	7	18	
77%	12	5	17	
90%	1	0	1	

The frequency of visits by the geriatric patients to the emergency department within three months before admission was evaluated. It showed a mean value of 3.69 (standard deviation, 4.209). For 36 patients, it was their first visit to the ED. Sixty-five patients had more than one visit to the ED, of which 20 visited our hospital, while 45 visited different hospitals. Fifty-two patients revisited the ED because of complaints similar to those presented at their previous visits. Thirteen patients revisited the ED because of other complaints.

## Discussion

Overcrowding of the ED by the geriatric population has become a challenge worldwide. Improvements have been made in the healthcare sector in Pakistan [10]. However, geriatric care is still somewhat lacking, especially in the ED setting; EDs are not well equipped to handle geriatric patients. The lack of specialist geriatric healthcare workers hampers not only the ED but also other departments. Moreover, the ED setting is not designed for high-quality geriatric care. Patients waiting for diagnostic tests may lie for hours in stretchers, risking pressure ulcers under glaring overhead lights in a windowless environment, which may lead to disorientation. Furthermore, the ED’s floors are of hard vinyl or marble that is easy to clean but can be a fall hazard for geriatric patients [11]. 

Approximately half of the study patients were in the 65-70 years age group. This contrasts with research in the USA [12]. Our different results may be due to Pakistan’s higher mortality rate, which is due to malnutrition and poverty [2]. Because a large number of Urdu-speaking communities live in urban areas, especially in Karachi, there were more Urdu-speaking patients than other ethnicities. Almost all of the patients were living with their families. This is consistent with the cultural norm in Pakistan, where elderly parents live with their families, especially those who are suffering from illness. 

It is very time-consuming to diagnose geriatric patients. Even then, misdiagnosis is more likely, which may result in discharging patients with unrecognized and untreated health problems [13,14]. Most of our study patients suffered from gastrointestinal disorders. This might be due to the high prevalence of hepatitis C (4.8%) in the Pakistani population-the second-largest affected population [15]. Hooker et al. also concluded that ED visits were most commonly due to gastrointestinal symptoms [16]. 

Frailty is very common in elderly individuals, increasing their risks of disability, morbidity, and mortality. More than half of the study patients were found to be frail. This is in concordance with a study that used different frailty screening instruments [17]. The comorbidity index was higher in the frail group compared with the pre-frail and robust groups, which is also consistent with previous literature [8]. 

Industrialization and urbanization have led to changes in occupations and lifestyles that increase the risks of hypertension and diabetes [18]. These diseases are already increasing in the aging population, as suggested by a previous study [19]. Because of their chronic nature, diabetes and hypertension have higher risks of disease progression in the elderly, which may explain the high numbers of patients who report vision loss, cerebrovascular episodes, and cognitive impairment [20]. High proportions of elderly patients with hypertension and/or diabetes were found to experience cerebrovascular episodes. 

It was found that patients with a history of falls were likely to have poor outcomes resulting in revisits to the ED. A previous study reached the same finding [21]. The CCI helped us to predict the ten-year survival rate of the patients. It was found that almost a quarter of patients had a zero percent 10-year survival rate. This accords with the life expectancy in Pakistan (67.273 years) [22]. 

Almost two-thirds of the study patients revisited the ED. We explored associations between frailty and re-visits to the ED. It was found that frailty was strongly associated with readmission: the frailer a patient, the greater their likelihood of revisiting. This finding is supported by a previous study [23]. 

Many revisits occurred because of a lack of specialists in geriatrics or a lack of resources and facilities. Furthermore, the misbehavior of elderly patients can lead to their mismanagement and, sometimes, misdiagnosis.

Limitations

This was a cross-sectional study with very small sample size. Therefore, most of our results are not statistically significant. There was also a very short time for data collection. As a single-center study, we may have obtained results that would differ in other EDs. Further prospective, multi-center studies are required. Also, comorbidities were reported by the patients’ attendants or caregivers; there may have been an error in this reporting.

## Conclusions

It was concluded that the need for geriatric care is worth mentioning since it imposes a significant burden on the ED. The geriatric patients had a higher risk of being on the critical list as most of them were frail and suffered from multiple severe comorbidities. Improvement of emergency care for the elderly will almost certainly necessitate interdisciplinary initiatives to assist healthcare workers, as well as necessary, evidence-based structural and operational modifications to the ED. Perhaps it is now time to address the unique requirements of geriatric patients in the ED. It is suggested that implementing geriatric ED interventions can feasibly target incremental improvements in patient care in EDs, not only in terms of the physical space but also of the treatment methods.
